# Persistent cough and asthma-like symptoms post COVID-19 hospitalization in children

**DOI:** 10.1186/s12879-022-07252-2

**Published:** 2022-03-12

**Authors:** Hossein Esmaeilzadeh, Anahita Sanaei Dashti, Negar Mortazavi, Hossein Fatemian, Mohebat Vali

**Affiliations:** 1 Allergy Research Center, Shiraz University of Medical Sciences, P. O. Box 719335899, Shiraz, Iran; 2 Department of Allergy and Clinical Immunology, Namazi Hospital, Shiraz University of Medical Sciences, Shiraz, Iran; 3 Alborzi Clinical Microbiology Research Center, Shiraz University of Medical Sciences, Shiraz, Iran; 4 Department of Clinical Pharmacy, School of Pharmacy, Shiraz University of Medical Sciences, P. O. Box 719335899, Shiraz, Iran; 5 Student Research Committee, Shiraz University of Medical Sciences, Shiraz, Iran

**Keywords:** COVID-19, Hospitalization, Asthma, Child

## Abstract

**Backgrounds:**

Respiratory viruses are the main triggers of asthma. Coronavirus is shown to contribute to respiratory tract infections that can lead to prolonged cough and asthma.

**Objectives:**

Present study aimed to determine the risk of developing Persistent cough and asthma-like symptoms in hospitalized children due to COVID-19.

**Methods:**

This prospective study was carried out in a tertiary referral center. During the COVID-19 pandemic, 69 hospitalized pediatric patients admitted with COVID-19 were observed from February 2020 to January 2021. Clinical and laboratory data were recorded, and after discharge, patients were followed and visited for cough and asthma evaluation one, 2 and 6 months later. Patients with asthma-like diagnoses in follow up defined as asthma-like groups, and patients without any sign of asthma were categorized as the non-asthma group. Asthma-like co-morbids and risk factors were evaluated and compared between the two groups.

**Results:**

In follow-up, most of the COVID-19 hospitalized patients (N = 42) (58.5%) were not affected by asthma-like symptoms. 60.9% of the COVID-19 patients were male. The asthma-like group cases had a significantly familial history of asthma (63.0%), past medical history of asthma (33.3%), and Allergic rhinitis (85.2%). Rates of signs and symptoms during hospitalization were significantly higher in patients with COVID-19 and past medical history of asthma.

**Conclusions:**

We found an asthma-like prevalence of 41.5% in the cohort of COVID-19 hospitalized children. Family history of asthma and previous history of asthma and allergic rhinitis are risk factors for asthma-like after COVID-19 hospitalization. COVID-19 presentations are more severe in the asthma-like group.

## Background

As a new beta coronavirus, the severe acute respiratory syndrome coronavirus two was first identified in December 2019. Coronavirus disease 2019 (COVID-19) rapidly spread worldwide, characterized by severe pneumonia and other complications, such as death in highly severe cases. The diseases spread rapidly in the community due to the easy transmission of the virus, even from asymptomatic patients. Moreover, the causing agent survives in respiratory droplets and fomites [1]. Three months following the first emergence, about 2.6 million cases were reported globally due to high community transmission. Some comorbidities exacerbate COVID-19 outcomes: hypertension, chronic obstructive pulmonary disease, diabetes mellitus, cardiovascular disease, obesity, and asthma [2, 3].

In the United States, asthma is among the most prevalent chronic diseases affecting 8–9% of the population, and its acute exacerbations are considered as a common reason for hospitalizations and/or visits to the emergency rooms [4]. Respiratory viruses are usually regarded as asthma triggers [5–7]. Therefore, coronaviruses, which are respiratory viruses, have been shown to contribute to the infections of the respiratory tract and asthma exacerbations [8].

Currently, asthma risk factors in children induced by COVID-19 are not clear. The studies performed in China and the United States reported ≤ 1% and 7.4–17% prevalence for asthma in patients with COVID-19, respectively [9, 10]. Another study claimed the asthma prevalence of 1.82% among COVID patients [11].

The CDC currently classifies uncontrolled moderate to severe asthma as a high-risk group susceptible to severe COVID-19. The signs of COVID-19 in patients with asthma include cough, breath shortness, and chest tightness. The differentiation of these symptoms from severe asthma exacerbation is difficult. It is especially true about children with a lower ability to recount their signs and possibly varying symptoms than adults are.

On the other hand, it has been demonstrated that viral infections, specially severe forms that require hospitalization, activate immunological mechanisms and induce morphological changes such as tissue remodelling that can contribute to the initiation or aggravation of asthma [12, 13]. It is stated that while allergen sensitivity is one of the strongest risk factors for asthma, it rarely directly leads to persistent asthma. Instead, allergies often cause asthma in conjunction with other pre-inflammatory environmental factors, especially respiratory viral infections [14].

Consequently, the present study aimed to describe the association of asthma-like symptoms in hospitalized children affected by COVID-19. In addition, we examined whether hospitalization because of COVID-19 might result in the development of persistent cough and asthma-like following COVID-19.

## Materials and methods

### Identifying patients with COVID-19

This prospective study was carried out in Namazi tertiary referral Hospital, Shiraz, as the largest hospital in the south of Iran. Patients were identified based on a specialist physician visit. Patient information is extracted by reviewing the file data, an electronic repository of the health records of hospitalized patients in the health system. Shiraz University of Medical Sciences, with the number 23556-35-01-99, approved the current study, and the authors took consent from the patient's parents or caregivers.

The inclusion criteria entailed the age of < 18 years old, being evaluated from February 2020 to January 2021 in Shiraz, and having received the diagnosis code of COVID-19 (U07.1) according to the Tenth Revision of the International Classification of Diseases (ICD-10). Presumed COVID-19 patients (U07.2) whom RT-PCR did not confirm were excluded. Moreover, death, the lack of response to call or avoid participating in this cohort study, are affected by other infectious diseases, such as pneumonia, post-infectious cough in 2 months after infection, and patients considered COVID-19 outpatients were the other exclusion criteria.

### Asthma-like diagnosis in patients with COVID-19

**Fig. 1:**
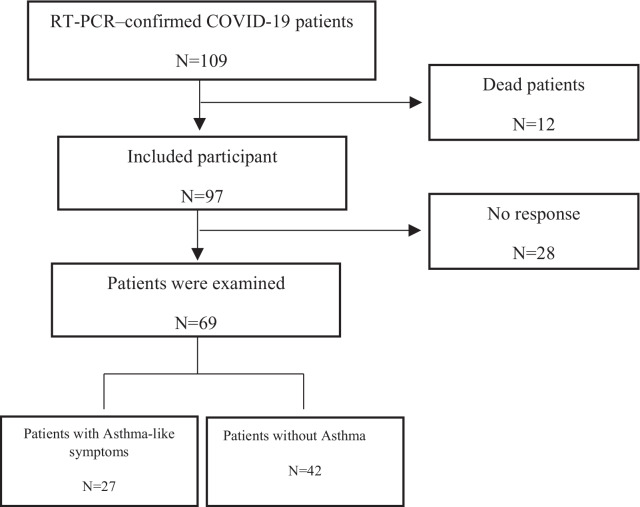
Algorithm of identifying COVID-19 or asthma-like patients

Data of 109 COVID-19 admitted patients confirmed by RT-PCR were collected. Twelve of the subjects died and were excluded from the study. The rest of the patients visited one and two months after recovery and were discharged, then followed monthly until six months later. We followed the patients by clinic visit at 1, 2 and 6 months and by phone in other months, and if necessary, the patients asked to come to the clinic. Twenty-eight participants did not respond to our call or did not refer and were excluded from the study resulting in 69 patients (Fig. 1).

Patients requested to refer to the clinic for asthma-like diagnosis for medical history and clinical examination. In addition, the PFT test was performed for children aged over five years old. The diagnostic criteria of the Gina guideline [15] included coughs that cause the person to wake up, coughs when exercising or laughing, frequent dry coughs during the day, and changes in PFT in patients over five years old. In PFT, asthma-like was diagnosed if FEV1/FVC was < 80% and elevated by 12% in FEV1 of 200 cc following the administration of a bronchodilator. Coughs behind two months registered as a persistent cough.

### Identification of clinical characteristics and comorbidities

Clinical characteristics include age, gender, familial history, history of asthma, and Allergic rhinitis and signs evaluated. The symptoms during hospitalization were assessed as a checklist, including fever, cough, dyspnea, sputum, myalgia, headache, diarrhea, vomiting, abdominal pain, cardiac complication, dry cough, waking cough, activity cough, chest tightness, chest wheezing, weight loss, nasal congestion, and smell decrease. Moreover, laboratory test results, past medical history, and the patient's family history regarding asthma and other allergic diseases were recorded.

### Statistical analysis

Demographic characteristics and clinical data were evaluated for all the patients and compared by the chi-square test. The laboratory test results were compared utilizing the Mann–Whitney test. The correlation between asthma-like and COVID-19 hospitalization was examined in these patients.

## Results

### Asthma-like prevalence among COVID-19 patients

One hundred nine patients were identified with an ICD-10 diagnosis code of COVID-19 based on patients' medical records referred from February 2020 to January 2021. The following results related to the study of patients two months after discharge. One month after discharge, 30 patients had symptoms, and it seemed to mostly post-infectious of COVID19, and in the 2th month, 20% had no symptoms. During the six months following, no new patient was added. Out of 109 patients, 97 (88.9%) cases were confirmed by RT-PCR and were included in the study. Finally, 69 patients were analyzed, excluding those who died or did not respond (Table 1). Most of the COVID-19 patients (N = 42) (58.5%) were not affected by post-hospitalization asthma-like. Among 27 patients with post-COVID-19 asthma-like, 14 (51.9%) participants were age > 5 years old and confirmed as asthma-like using a spirometer (PFT) (Fig. 1). Of the patients, 15.94% had a history of asthma at the time of admission (Table 1), of which 55% had a previous history of childhood asthma that had been stopped medications, and the rest received asthma medications, with 20% having moderate persistent asthma and 30% mild persistent asthma. Also, the patients we diagnosed with asthma-like after discharge had a 20% history of the previous hospitalization for asthma attacks, of which only about 45% had active asthma at the time of admission and were taking medication. The rest were patients who had stopped taking their medications.

Among those with a history of previous asthma with discontinuation of the drug, one (20%) showed worse asthma symptoms. Of the remaining people with current active asthma who were taking medication, 2 (50%) had worsening symptoms. Of the 12 people who were expired and did not enter our study, one (8.33%) had active asthma and was taking medication, and the severity of symptoms worsened in this person.

### Demographic and clinical characteristics of COVID-19 patients with and without post-hospitalization asthma-like

We evaluated and compared diverse demographic characteristics and clinical data between COVID-19 patients with and without post-hospitalization asthma-like (Table 1). Most (37.3%) COVID-19 patients were < 5 years, regardless of asthma-like status. Slightly over half (60.9%) of the COVID-19 patients were male. The asthma-like group cases had a very high familial history of asthma (63.0%), past medical history of asthma (33.3%), and Allergic rhinitis (85.2%).

### Clinical comorbidities of COVID-19 patients with and without post-hospitalization asthma-like

**Fig. 2:**
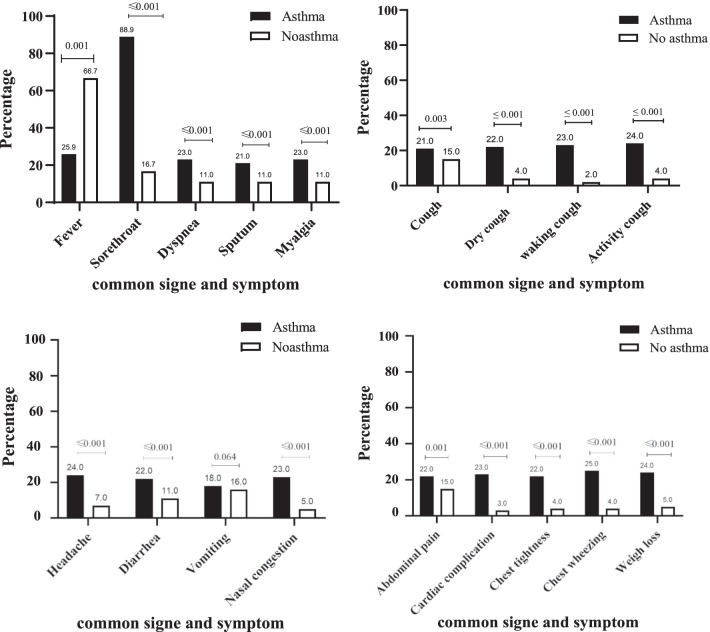
Prevalence of signs and symptoms during hospitalization in COVID-19 patients stratified based on asthma-like status; A total of 69 patients were investigated

Afterward, we specified the prevalence of diverse comorbidities and symptoms in COVID-19 patients based on post-hospitalization asthma-like status (Fig. 2). Rates of symptoms were significantly elevated in the group of patients with both COVID-19 and asthma-like compared to COVID-19 patients without asthma. However, fever was found to be higher in the asthma-like group, and vomiting was not significantly different between patients with and without post-hospitalization asthma-like (Fig. 2).

Frequency of symptoms was not present among those who initially had asthma and patients who developed asthma after Covid 19 (fever: 25.9 vs., 22.1, P value = 0.822 & sore throat: 88.9 vs. 85.6, P value = 0.794 & Dyspnea: 23.0 vs. 20.5, P value = 0.877 & Sputum: 21.0 vs. 20.6, P value = 0.979 & Myalgia: 23.0 vs. 21.5, P value = 0.926 & cough: 21.0 vs. 19.8 P value = 0.939 & Headache: 24.0 vs. 22.7, P value = 0.937 & Diarrhea: 22.0 vs. 19.9, P value = 0.895 & Vomiting: 18.0 vs. 17.8, P value = 0.989).

### Laboratory data at COVID-19 diagnosis with post-hospitalization asthma-like status

**Fig. 3:**
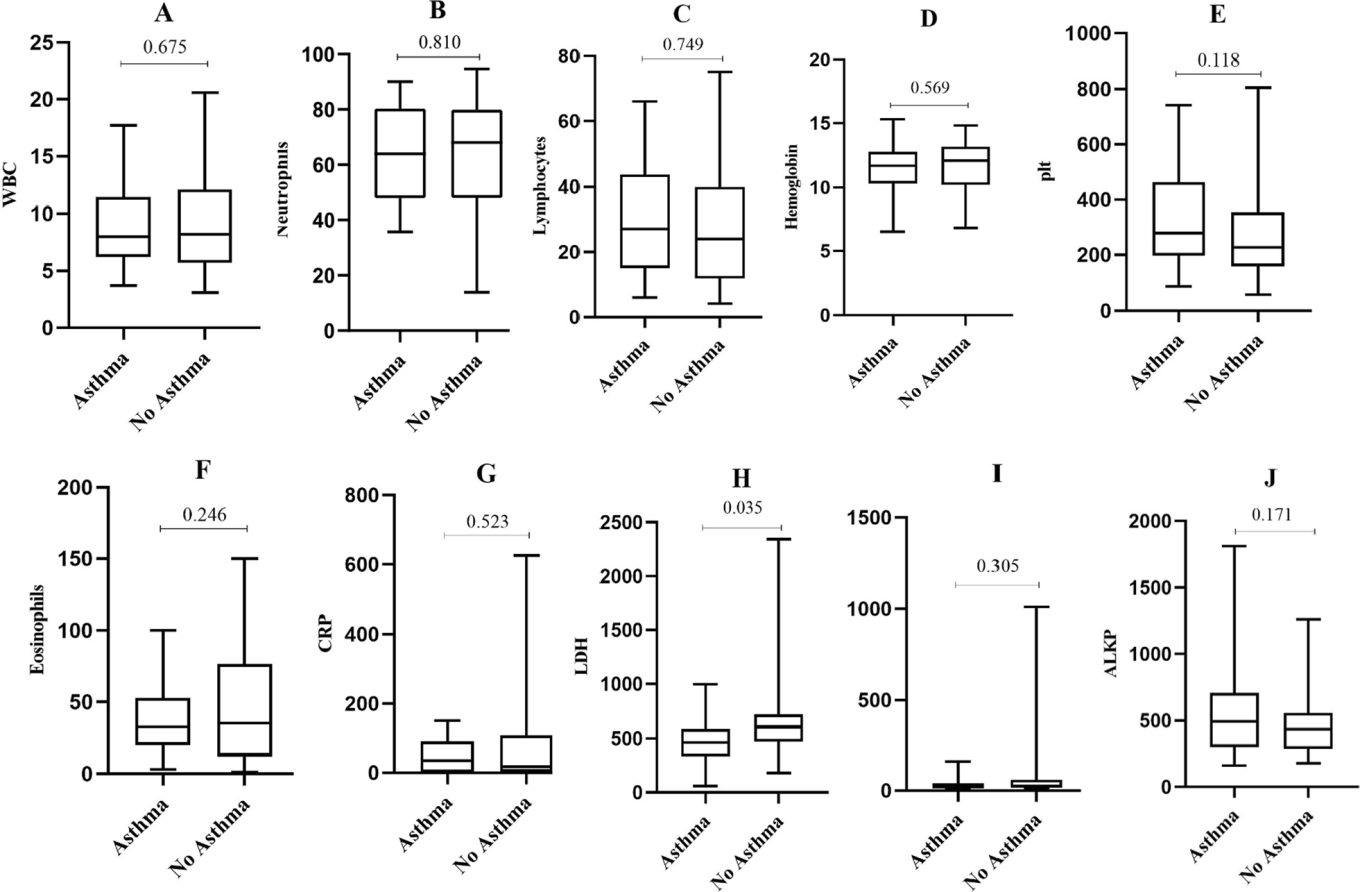
Laboratory results at COVID-19 diagnosis in hospitalized patients with asthma-like, in comparison with patients without asthma. The box is extended from percentiles 25th to 75th. The line in the box represents the median with ‘‘1’’ demonstrated as the mean. Whiskers are the minimum and maximum values. The nonparametric Mann–Whitney 2-tailed test was applied for statistical analysis. **A**: White Blood Cells (WBC); **B**: Neutrophils; **C**: Lymphocytes; **D**: Hemoglobin; **E**: Platet; **F**: Eosinophils; **G**: C-Reactive Protein (CRP); **H**: Lactate Dehydrogenase (LDH); **I**: Alanine transaminase (ALT); **J**: Alkaline phosphatase (ALKP)

We collected the results of different laboratory tests for all hospitalized cases when they were diagnosed with COVID-19 (Fig. 3). Complete blood cell count indicated that White Blood Cell (WBC), Hemoglobin (Hb), Placket (plt), C-reactive Protein (CRP), Alanine Aminotransferase(ALT), Alkaline phosphatase (ALkP), and eosinophil count were not significantly different between patients with and without post-hospitalization asthma-like (Fig. 3).

## Discussion

This is the first comprehensive cohort study on children with COVID-19 and post-hospitalization asthma-like to the best of our knowledge. 41.5% of COVID-19 patients had asthma-like after discharge in the present study. Our study population had a higher prevalence of persistent cough and asthma-like symptoms than the US and Chicago populations, which were estimated to have an asthma prevalence of 8–9% and 9.5%, respectively, at all [4, 16]. Moreover, recently published papers in the US reported a prevalence of 7.4–17% for asthma in hospitalized patients with COVID-19 [2, 17–19]. The latter reports are contrary to the low asthma prevalence of ≤ 1% observed in China [9, 10]. These heterogeneous findings could be attributed to genetic, geographically different asthma, screening time (during hospitalization or post-discharge asthma), and frequency or techniques for ascertainment. Given the higher percentage of asthma reported in our study than in other studies, it seems that since most previous articles have screened for asthma during hospitalization since we have examined a longer distance, it turns out that asthma can be a reaction and a delay event after COVID-19, especially in children.

Symptoms, which are clearly established to be associated with COVID-19, existed in patients with post-hospitalization asthma-like (Fig. 2). These symptoms include fever, cough, dyspnea, sputum, myalgia, headache, diarrhea, vomiting, abdominal pain, cardiac complication, dry cough, waking cough, activity cough, chest tightness, chest wheezing, weight loss, nasal congestion, and smell decrease Which were significantly higher in the post-covid19 asthma-like group. Except for vomiting, which was not significantly different between patients with and without post-hospitalization asthma-like. In addition, Laboratory results demonstrated that WBC, lymphocyte, neutrophil, plt, Hb, ALT, eosinophil, CRP, and ALKP were not significantly different between patients with and without post-hospitalization asthma-like.

Previous studies [20] have shown that a decrease in lymphocytes and an increase in CRP can predict the chances of hospitalization, since the two factors are statistically different in hospitalized patients with asthma-like, in comparison with patients without asthma no significance was seen (0.810 and 0.523, respectively). Therefore, we found that asthma was not a risk factor for admission and hospitalization in COVID-19 patients. We also found that post-discharge asthma-like was higher in patients with a previous history of asthma than in patients without it, and this difference was statistically significant. Therefore, it seems that the background of asthma can be a risk factor for post-discharge asthma-like in COVID19 patients. According to a recent report, despite the high incidence of asthma, these chronic respiratory conditions have not been reliably reported as major comorbidity for COVID-19 [1]. The evidence presented in this study contradicts the information provided by Lippi and Henry [21] that Severe respiratory disease is associated with severe forms of COVID-19. Such a discrepancy could be because there is no consensus on the severity of COVID-19 patients and all the features of acute respiratory distress syndrome (ARDS) instead of acute lung injury (ALI) [22–24]. It may be difficult or impossible for non-intubated people. However, hospitalized COVID-19 patients with asthma are expected to perform worse than patients without. Because SARS-CoV-2 uses the angiotensin-converting enzyme (ACE-2) as a cellular receptor that is suspected to be higher in obstructive respiratory disease, so expected that these individuals would be at higher risk for hospitalization [24]. In fact, reducing the risk of hospitalization in patients with asthma and COVID-19 could be associated with the use of inhaled corticosteroids (ICS), which have recently been shown to have a protective effect against infections, specifically those due to coronaviruses [25].

Therefore, asthma appears to be not a primary risk factor to increase the chance of developing COVID19 or, more severely, admission unless the asthma is uncontrolled. However, background asthma causes recurrent asthma after discharge from the hospital. The above findings can probably also be explained as phenomenon of ‘postviral hypereactive airways’ seen commonly in pulmonary viral infections as well as viral infection induced exacerbations of previous asthma.

Our study had several limitations. Some data were collected retrospectively after the patient's discharge, causing limitations for drawing associations rather than causal inferences. Another limitation of the current study was that we assessed the data collected from February 2020-January, 2021, and the findings may alter as the result of collecting more data after the study period. It assumed that the possibility of testing patients affected by asthma was higher due to the chronic nature of this lung disease. Another limitation is that we investigate the development of asthma in hospitalized COVID-19 patients with more severe forms of infection compared to outpatients, which does not represent all COVID-19 patients. Low sample size is another limitation that make it difficult to globalize the result.

## Conclusions

We found a post-hospitalization persistent cough and asthma-like prevalence of 41.5% in the cohort of COVID-19 hospitalized patients. Our study indicates that post-hospitalization asthma-like risk factors may have a family history of asthma and previous history of asthma and Allergic rhinitis. The incidence of COVID-19 signs and symptoms is higher in the asthma-like group, especially fever. However, no difference was observed between the two groups in terms of laboratory information. It is necessary to assess post-hospitalization asthma-like in children diagnosed with COVID-19.

**Table 1:** Demographic and clinical characteristics of patients with COVID-19 confirmed by RT-PCR and stratified by asthma-like status

Characteristic		All patients	No asthma	Asthma-like	P value
N (%)		69 (100)	42 (58.5)	27 (41.5)	
Age(year)					
< 6		28 (41.8)	15 (35.7)	13 (48.1)	0.332
6–12		28 (41.8)	20 (47.6)	8 (29.6)	
12–18		13 (16.4)	7 (16.7)	6 (22.2)	
Sex					
Female		27 (39.1)	16 (38.1)	11 (40.7)	0.826
Male		42 (60.9)	26 (61.9)	16 (59.3)	
Family history of asthma					
Yes		18 (26.1)	1 (2.4)	17 (63.0)	≤ 0.001
No		51 (73.9)	41 (97.6)	10 (37.0)	
History asthma					
Yes		11 (15.94)	2 (4.76)	9 (33.33)	0.0016
No		58 (84.06)	40 (95.24)	18 (66.67)	
Family history of Allergic rhinitis					
Yes		26 (37.7)	3 (7.1)	23 (85.2)	≤ 0.001
No		43 (62.3)	39 (92.9)	4 (14.8)	
Allergic rhinitis					
Yes		28 (40.6)	5 (11.9)	23 (85.2)	≤ 0.001
No		41 (59.4)	37 (88.1)	4 (14.8)	

## Data Availability

The data supporting this study's findings are available on request from the corresponding author, [HE]. The data are not publicly available due to containing information that could compromise the privacy of research participants.

## Abbreviations

COVID‐19: Coronavirus disease 2019
CDC: Centers for Disease Control and Prevention
ICD: International Classification of Diseases
PFT: Pulmonary Function Tests
WBC: White blood cell
Hb: Hemoglobin Concentration
plt: Platelet Count Test
ESR: Erythrocyte Sedimentation Rate
CRP: C—reactive protein
LDH: Lactate Dehydrogenase
ALT: Alanine transaminase test
ALKP: Alkaline Phosphatase
